# Anthocyanin Supplementation and Inflammation: A Systematic Review and Meta‐Analysis of IL‐8, IL‐10, IL‐18, IFN‐γ, and Resistin in Healthy, Overweight, and Obese Populations

**DOI:** 10.1002/fsn3.71527

**Published:** 2026-02-12

**Authors:** Alessandro Young, Madiha Ajaz, Lada Vugic, Natalie Shilton

**Affiliations:** ^1^ School of Pharmacy and Medical Sciences Griffith University Gold Coast Queensland Australia

**Keywords:** anthocyanin, inflammatory markers, meta‐analysis, obesity

## Abstract

Previous research has demonstrated the anti‐inflammatory effects of anthocyanin supplementation, as evidenced by reduced levels of inflammatory adipokines in obese populations. However, the relationship between anthocyanin intake and obesity‐related adipokines remains unclear in existing research. To investigate this, we hypothesised that anthocyanin supplementation would significantly reduce plasma concentrations of interleukin‐8 (IL‐8), IL‐18, interferon‐gamma (IFN‐γ) and resistin, and significantly increase plasma concentrations of IL‐10 in a healthy, overweight, and obese population. A systematic search of PubMed and Scopus was conducted between September 2024 and November 2024, resulting in 22 eligible studies for inclusion. The standardised mean difference was used to establish the effect size, and I‐squared was used to determine heterogeneity. The risk of bias was assessed using Cochrane's risk of bias assessment tool. An Egger's test and funnel plot were utilised to determine any publication bias. The meta‐analysis data showed that the inclusion of anthocyanins resulted in significant reductions in IL‐8 (*p* = 0.004) and IFN‐γ (*p* = 0.02). However, we observed that IL‐10 (*p* = 0.97), IL‐18 (*p* = 0.28) and resistin (*p* = 0.42) did not show a significant effect after anthocyanin consumption. The findings indicate that anthocyanin supplementation can significantly decrease circulating plasma levels of IL‐8 and IFN‐γ in healthy, overweight, and obese populations, but not IL‐10, IL‐18 or resistin levels.

**Trial Registration:** PROSPERO: CRD420251037683 (https://www.crd.york.ac.uk/PROSPERO/view/CRD420251037683).

## Graphical abstract

This study presents a systematic review and meta‐analysis of anthocyanin supplementation (> 40 mg/day) and its effects on inflammatory cytokines in healthy, overweight and obese adults. Literature searches in PubMed and Scopus identified 22 eligible studies (*n* = 1299). Meta‐analysis showed significant reductions in IL‐8 and IFN‐γ, while IL‐10, IL‐18 and resistin were not significantly affected. Overall risk of bias was low, with no evidence of publication bias.

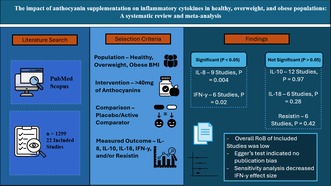

## 1 Introduction

The obesity epidemic presents a significant challenge in chronic disease prevention and health across the world. It contributes to the development of numerous chronic conditions, such as T2DM, high blood pressure and heart disease, making it one of the significant health challenges of the 21st century (Khanna et al. 2022). The World Health Organization illustrates that more than one billion people are classified as obese (body mass index [BMI] of > 30), of which four million have died as a result (Laurence 2024). The economic impacts of this epidemic have led to 3 trillion USD (approximately 4.6 trillion AUD) and more than 18 trillion by 2060 (Okunogbe et al. 2021). One of the major causes of this epidemic is the easy access to highly palatable, ultra‐processed, high‐sugar and high‐fat foods that are consumed daily. Western‐style diets typically consist of high‐sugar, high‐carbohydrate, and unhealthy fat foods, resulting in a calorie surplus. Excessive food intake stimulates adipose tissue to release an excessive number of inflammatory mediators. Obesity is a significant predictor of metabolic disorders due to the increased development of insulin‐resistant adipocytes and the activation of macrophages (Khanna et al. 2022; Williams et al. 2017). This is due to the upregulation of pro‐inflammatory adipocytokines from excess macronutrients, leading to adipose tissue hypertrophy and death (Huang et al. 2022; Ouchi et al. 2011; Rohm et al. 2022). Adipocytes secrete hormones named adipokines that regulate the metabolism of multiple organs. This causes the development of T2DM and other common inflammatory conditions, including cardiovascular disease (CVD) and hypertension, that are prevalent in one in three Australians.

**FIGURE 1:**
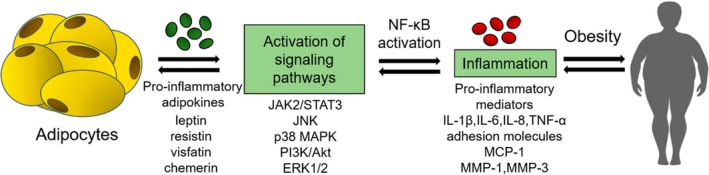
Representation of obesity‐induced inflammation, and the pro‐inflammatory effects of adipokines (Kirichenko et al. 2022).

Current medications that are directed to improve weight loss, such as Contrave and Semaglutide, have proven to work long‐term, but can lead to several side effects and are expensive, making them inaccessible to much of the population (Deng et al. 2022; Sherman et al. 2016; Wilding et al. 2021). Recent studies on the use of anthocyanins have found both anti‐inflammatory and potential anti‐obesity benefits. Foods such as strawberries, blueberries and purple potatoes are rich in anthocyanins and have been linked to reducing weight and body fat percentage when added to a healthy diet (Ulaszewska et al. 2020; Zhang et al. 2019). All types of anthocyanins have multiple mechanisms of action, including the capture of free radicals and the inhibition of low‐density protein (LDL) oxidation. Studies on the clinical use of these compounds suggest that they reduce inflammation by targeting PLA2, a group of esterases that generate arachidonic acid and free fatty acid (FFA) precursors in inflammatory pathways, thereby increasing the production of pro‐inflammatory cytokines in fat cells (Kozlowska and Dzierzanowski 2021; Ma et al. 2021). They also inhibit the activation of COX‐2 and NF‐κB signalling pathways, which have mechanisms of action similar to those of current anti‐obesity medications (Kozlowska and Dzierzanowski 2021; Ma et al. 2021). NF‐kB directly influences the transcription of many pro‐inflammatory cytokines, including IL‐8, IL‐6 and tumour necrosis factor‐alpha (TNF‐α) (Figure 1; Caamano and Hunter 2002). These cytokines are activators of NF‐kB, establishing a feedback loop that sustains chronic low‐grade inflammation. It also regulates the NLRP3 inflammasome axis, which is primed in clinical trials investigating obesity‐induced inflammation. Excess consumption of nutrients and metabolic stressors provides the activation signal, leading to the enhanced secretion of inflammatory cytokines (Mao et al. 2025).

Previous clinical trials have investigated the efficacy of anthocyanin supplementation on several adipokines, including IL‐8, IL‐18, IFN‐γ, IL‐10 and resistin, all of which directly influence the inflammatory response triggered by obesity (Bruun et al. 2007; Graf et al. 2013; McGillicuddy et al. 2009; Straczkowski et al. 2002). Pro‐inflammatory adipokines are elevated in obese and overweight individuals, serving as an accurate indicator of the effect of an anti‐inflammatory drug. These cytokines are associated with systemic inflammation and insulin resistance and exacerbate obesity‐related complications (Bruun et al. 2007; Graf et al. 2013; McGillicuddy et al. 2009; Straczkowski et al. 2002). Fewer studies have primarily measured these cytokines compared to more commonly measured cytokines, such as TNF‐α and IL‐6, due to their well‐characterized roles in obesity‐related inflammation (Ellulu et al. 2017). Despite the lack of studies on clinical implications, it should not undermine the significance of cytokines such as IL‐18 and resistin in obesity. This literature gap highlights the direction for a focused analysis of these adipokines. On the other hand, anti‐inflammatory cytokines, such as IL‐10, have an inverse relationship with obesity and are often dysregulated in this condition (Charles et al. 2011). Increased levels improve insulin sensitivity and prevent tissue inflammation and CVD. Indications of increased IL‐10 levels in obesity can show promising signs of anti‐inflammatory benefits.

The relationship between anthocyanins and inflammatory markers remains unclear; however, several studies have measured these markers in various populations and weight groups by comparing the intervention with a placebo or control group. Many meta‐analyses and systematic reviews have been conducted, including those by Song et al. (2023) and Xu et al. (2021), which analysed the effects of anthocyanin supplementation on IL‐6, TNF‐alpha, CRP, and VCAM‐1. However, few studies have investigated the effect of anthocyanins on IL‐8, IL‐10, IL‐18, IFN‐γ or resistin in healthy, overweight or obese populations. Most meta‐analyses have also not established the link between these biomarkers in human clinical trials or compared the differences in intervention and control groups across multiple BMI groups. This meta‐analysis aims to quantitatively evaluate the impact of anthocyanins on obesity‐induced inflammation by analysing clinical studies that assess the changes of specific adipocytokines (IL‐8, IL‐18, IFN‐γ, IL‐10 and resistin) across healthy, overweight and obese populations.

## 2 Methodology

### 2.1 Search Methods

Search strategies were conducted in accordance with the Preferred Reporting Items for Systematic Reviews and Meta‐Analysis (PRISMA) checklist provided as Table S1. The search was conducted between September 2024 and November 2024, utilising two electronic databases: PubMed and Scopus. Studies involved in the screening process are illustrated in Table 1. Studies were imported into Covidence for the screening process and to identify duplicates between databases. The search terms for both electronic databases were:

**TABLE 1:** Key characteristics of each included study.

Author(s)	Year	Sample size (*N*)	BMI group	Anthocyanin intervention	Dosage and duration	Comparison group	Adipocytokines measured	Key findings	Study design
Basu	Basu et al. 2021	45	Obese/pregnant (BMI > 30)	Blueberries and soluble fibre	700 mg/280 g daily for 18 weeks	No intervention	Resistin	Decrease in intervention and Control Group	RCT
Borda	Borda et al. 2024	201	Overweight (BMI > 25)	Anthocyanin capsule	320 mg daily for 24 weeks	Placebo	IL‐8, IL‐10, IFN‐γ	Decrease in IL‐8 and IFN‐γ, increase in IL‐10	RCT, Parallel Group
Chong	Chong et al. 2024	30	Overweight (BMI > 25)	DailyColorsTM	150 daily for 7 days	Placebo	IL‐8, IL‐10	decrease in IL‐10 and increase in IL‐8	RCT, crossover
Cremonini	Cremonini et al. 2022	24	Healthy	CDRE (cyanidin and delphinidin‐rich extract)	320.4 mg/g once	Placebo	IL‐18, IL‐8	Increase in IL‐8, No effect in IL‐18	RCT, crossover
Guo	Guo et al. 2020	111	Healthy	Anthocyanin capsule	40‐320 mg daily for 14 days	Placebo	IL‐10	Increase in IL‐10	RCT, Double‐Blind
Hokayem	Hokayem et al. 2013	43	Overweight (BMI > 25)	Grape polyphenol	86 mg daily for 9 weeks	Placebo	IFN‐γ, resistin	Decrease in IFN‐γ and resistin	RCT, Parallel Group
Karlsen	Karlsen et al. 2007	120	Healthy, overweight (BMI > 25)	Bilberry and black currant	300 mg, 2 capsules daily for 3 weeks	Placebo	IFN‐γ, IL‐8, IL‐10	Decrease in IFN‐γ, IL‐8 and IL‐10	RCT, Parallel Group
Kuntz	Kuntz et al. 2014	30	Healthy	Anthocyanin‐rich beverage	840‐983 mg/mL daily for 14 days	Placebo	IL‐8, IL‐10	Decrease in IL‐8, no effect in IL‐10	RCT, crossover
Lee	Lee et al. 2016	63	Overweight (BMI > 25)	Anthocyanin‐rich black soybean testa extracts (BBT)	12.58 mg, 2 capsules daily for 8 Weeks	Placebo	IL‐10	Increase in IL‐10	RCT, Parallel Group
Mendes	Mendes et al. 2021	20	Healthy	Jucara fruit juice	186 mg daily	No intervention	IL‐8, IL‐10	Decrease in IL‐10, no effect in IL‐8	RCT, crossover
Nikbakht	Nikbakht et al. 2021	14	Obese (BMI > 30)	Anthocyanin capsule	320 mg daily for 4 weeks	Healthy Group	IL‐8, IL‐18, resistin	Decrease in IL‐8, IL‐18 and resistin	Open‐label Study, Parallel Group
Ono‐Moore	Ono‐Moore et al. 2016	27	Healthy	HFM breakfast with blueberry powder	100 mg daily dose, then 2‐week washout period	No intervention	IL‐8	Decrease in IL‐8	RCT, crossover
Stote	Stote et al. 2017	19	Obese (BMI > 30)	Wild Blueberry Juice	314 mg/240 mL daily for 7 days	Placebo beverage	IL‐10	Increase in IL‐10	RCT, Crossover
Tome‐Carneiro	Tomé‐Carneiro et al. 2012	75	Overweight/obese	Resveratrol‐rich grape supplement	21.2/27.1 mg daily for 6 months	Placebo	IL‐10, IL‐18	Increase in IL‐10 and IL‐18	RCT, Parallel Group
Zhang	Zhang et al. 2020	176	Healthy/overweight	Anthocyanin capsule	40‐320 mg Daily for 12 Weeks	Placebo	IL‐10	No effect in IL‐10	RCT, Parallel Group
Zhu	Zhu et al. 2022	63	Overweight	Anthocyanin capsule	320 mg Daily for 12 Weeks	Placebo	IL‐18	Decrease in IL‐18	RCT, Parallel Group
Zunino	Zunino et al. 2014	24	Obese	Dietary grapes	14.16 mg/23 g Twice Da	Placebo powder	IFN‐γ, IL‐8, IL‐10	Decrease in IL‐8, increase in IL‐10 and IFN‐γ	RCT, crossover
Aboonabi	Aboonabi and Aboonabi 2020	40	Obese	Anthocyanin extract (MEDOX)	320 mg daily for 4 Weeks	Healthy Group	IL‐18	Decrease in IL‐18	Case–Control Study
Arbizu	Arbizu et al. 2023	60	Obese	Dark sweet cherry	70.21 mg twice/day for 30 days	Placebo	IL‐18, IL‐10, IFN‐γ	Decrease in IL‐18, IL‐10 and IFN‐γ	RCT, Single‐blind
Kim	Kim et al. 2018	37	Obese	Acai beverage	307 mg/L twice/day for 12 weeks	Placebo	IFN‐γ	Decrease in IFN‐γ	RCT, Double‐blind
Puupponen‐Pimia	Puupponen‐Pimiä et al. 2013	37	Obese	Ellagitannin‐rich Berries	70.7 mg daily for 16 weeks	No intervention	Resistin	Increase in resistin	RCT
Espinosa‐Moncada	Espinosa‐Moncada et al. 2018	40	Overweight/obese	Freeze‐dried Agraz	4.66 mg/g daily for 12 weeks	Placebo	Resistin	Increase in resistin	RCT, crossover

Keywords for Intervention; ‘anthocyanin’, ‘anthocyanins’, ‘anthocyanin‐rich’, ‘anthocyanins rich’, ‘dietary anthocyanin’, ‘anthocyanin supplementation’ or ‘anthocyanin extract’, ‘cyanidin’, ‘delphinidin’, ‘malvidin’, ‘peonidin’, ‘petunidin’, ‘pelargonidin’, ‘blueberry’, ‘bilberry’, ‘cranberry’, ‘blackcurrant’, ‘elderberry’, ‘grapes’, ‘raspberry’ and ‘pomegranate’.

Keywords for Outcomes; ‘inflammation’, ‘inflammatory’, ‘pro‐inflammatory’, ‘obesity’, ‘healthy’, ‘overweight’, ‘body weight’, ‘BW’, ‘BMI’, ‘body mass index’, ‘fat mass’, ‘waist circumference’, ‘waist‐hip ratio’, ‘IL‐8’, ‘interleukin‐8’, ‘IL‐18’, ‘interleukin‐18’, ‘resistin’, ‘IL‐10’, ‘interleukin‐10’, ‘IFN‐y’ and ‘Interferon gamma’.

Keywords for Study Design: ‘intervention’, ‘trial’, ‘random’, ‘randomized’, ‘randomised’, ‘RCT (randomised control trial)’, ‘randomized control trial’, ‘placebo’, ‘clinical trial’, ‘intervention studies’.

For optimizing search: Interleukin‐8, Interleukin‐10, Interleukin‐18, Interferon‐gamma and resistin.

**FIGURE 2:**
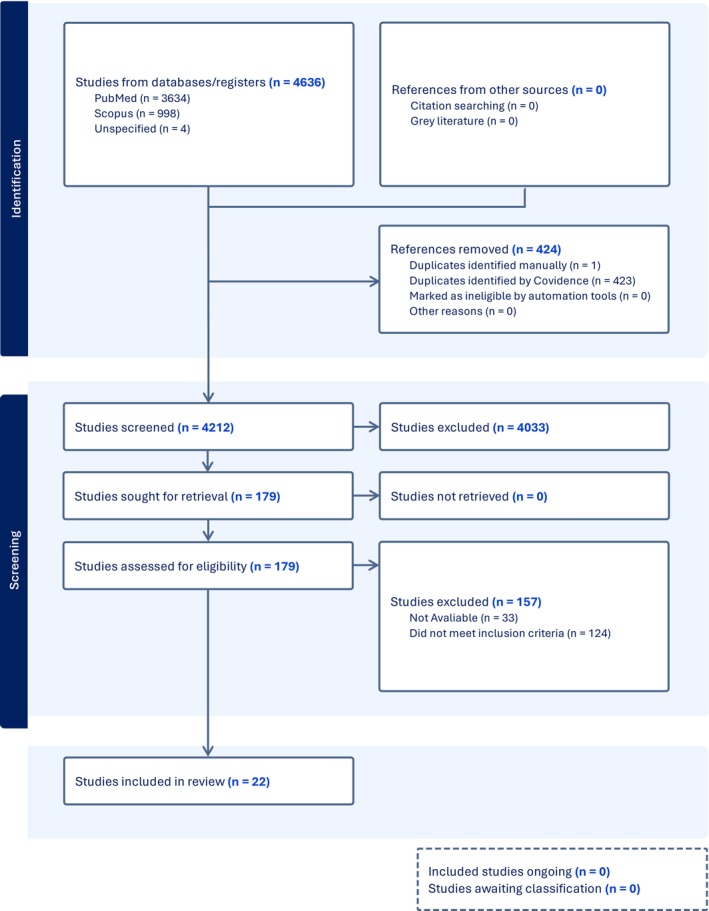
PRISMA flow diagram, representing the study identification, screening and inclusion results for a systematic review examining the effects of anthocyanin supplementation on inflammatory markers in multiple BMI groups.

A total of 4212 studies were screened for title and abstract screening, based on defined inclusion and exclusion criteria that were discussed beforehand. All screening was completed by two independent reviewers, AY and MA. Inter‐rater reliability was assessed using Cohen's kappa statistic. Following the initial screening, a full‐text review was conducted to determine whether the study was eligible for data extraction and statistical analysis. Both reviewers independently completed this stage of screening to reach a consensus on what data to include in the meta‐analysis. Any disagreements were handled through an assessment of the criteria. Initial and full‐text screening, as well as data extraction, were conducted using the Covidence software, which provides a user‐friendly and intuitive interface and features. Covidence also automatically identifies any duplicates and provides a PRISMA flowchart of all screening procedures, which includes justification for studies being excluded from the analysis and the reasons for their exclusion, which is illustrated in Figure 2. For any missing or additional data in the publication that is necessary for determining the outcome, reviewers will contact the author to request any needed information.

### 2.2 Study Selection Criteria

The eligibility of each study was determined using the PICO (Population, Intervention, Comparison, Outcomes) framework. All clinical studies were involved in the results of the meta‐analysis if they met the following criteria:

*Population*: Categorised into the three BMI groups, healthy (18.5–24.9 BMI), overweight (25–29.9 BMI) and/or obese (> 30 BMI), according to the WHO's BMI classification for adults. Studies using modified BMI categorisation, such as the WHO Asian‐Pacific criteria, are included, provided they state their classification system. BMI was selected as the sole indicator of adiposity, mainly due to its simple comparability across different studies. The study must specify when their BMI or weight was measured, and the method used for diagnosis. Studies that failed to report the BMI or classification of participants in each study arm were excluded from further screening. This focus on all BMI groups allowed for a comparison of the efficacy of anthocyanin supplementation across all BMI groups. It addressed how it may reduce low‐grade, chronic inflammation and improve fat loss at different stages of weight status. The inclusion of healthy BMI participants increased generalizability and increased the number of eligible studies, as there was a lack of research available. Furthermore, normal weight obesity has also been associated with inflammatory phenotypes (Mohammadian Khonsari et al. 2023). All participants were 18 years of age or older; individuals under 18 years of age were excluded from the study. Male and female participants were included, and studies that were single‐sex, as long as they maintained a focus on generalizability to a broader population. Studies that contained only one subset of relevant participants were included if they fell into the predefined categories and involved a comparison group.

*Intervention*: The primary intervention involved either an anthocyanin supplement or anthocyanin‐rich foods, such as berries, fruit extracts, or a combination of these. If the intervention was a supplement, the study stated whether it or the company produced the compound, and the contents of anthocyanins, as well as their concentration. If the intervention was a food, the study listed the berries or extracts it contains and the concentration of anthocyanins. Each study specified the dose that was being administered and the frequency of consumption during the trial. A minimum of 40 mg is required for inclusion, based on previous studies that have observed effective doses in reducing inflammation (Asgary et al. 2013; Zhang et al. 2020). Studies reporting results with multiple doses, their participants who consumed < 40 mg will not be included in the meta‐analysis. The duration of the pre‐ and post‐intervention periods was a minimum of 1 week or a duration sufficient to allow for an observational comparison.

*Comparison*: Eligible types of comparators included either a placebo, a control group receiving no intervention, or an active comparator. The placebo contents were stated, which may have consisted of the anthocyanin concentration. The frequency of these comparators matched the intervention frequency or was stated in the methodology. Active comparators were either different supplements or foods that have multiple concentrations of anthocyanins. If a control group is compared with various doses of anthocyanins, then all arm groups will be included in the intervention group during meta‐analysis.

*Outcome*: Each study included at least one of the adipokines (IL‐8, IL‐10, IL‐18, IFN‐γ, resistin) analysed in this study.

*Study model*: The following study designs were included for data collection; all studies were evaluated for risk of bias and academic integrity.

– Randomised Control Trials—Any blinding (open trial, single‐blind, double‐blind, triple‐blind) can also be parallel group or crossover trials.
– Quasi‐Randomised Trials—Included as there is an element of randomisation; however, they are prone to bias
– Observational Studies—Cohort and case–control studies are not recommended due to their high risk of bias and confounding factors; however, given the limited number of available studies, these studies will be considered.

All eligible studies included the location of the research, with the institution's name and address, and were published within the last 20 years. Only studies published in English were included, unless an English version of that study was available. To ensure that the information published upholds academic integrity and reliability, studies were either published in a peer‐reviewed journal or justified the quality of their methods and results.

### 2.3 Data Extraction and Quality Assessment

Studies were eligible for data extraction and quality assessment if they passed title/abstract and full‐text screening. Data were extracted and managed through two independent reviewers (AY and MA) for each study. Both reviewers conducted data extraction independently, with discussion limited to resolving outliers during the consensus phase. Covidence was used as it provides quality assessment tools for evaluating the risk of bias, and data extraction is implemented to manage the overall findings of each study. The quality of each study was assessed using Cochrane's risk of bias assessment tool. This provides an evaluation of the randomisation process, blinding of participants and personnel, missing outcome data, bias in the outcomes, selective reporting, or any other bias. Each category is marked as a low risk of bias, a high risk of bias, or unclear.

### 2.4 Statistical Analysis

RevMan (Review Manager) software was used to perform statistical analysis. The effect size was measured by calculating the mean difference with a 95% confidence interval; however, due to some studies using different scales of measurement, the outcomes were standardised using the standardised mean difference. Several studies reported results as the median or used standard error, in which the data were used to calculate the standard deviation, and the median was assumed to be the mean of the dataset. When changes from baseline means and standard deviations were not reported, they were obtained from the standard errors or confidence intervals provided by the study. It was calculated as recommended in the Cochrane Handbook, Section 6.5.2, through studies that did not provide the mean or standard deviation in the original trial (Julian et al. 2025). Participants across BMI categories were pooled together in the analysis. *p*‐Values or *Z*‐scores were used to evaluate the overall significance of the combined studies in each outcome. Heterogeneity was determined using Chi‐squared and I‐squared statistics, where 0%–40% signifies no heterogeneity, 40%–70% signifies moderate heterogeneity and 70%–100% signifies considerable heterogeneity. Sensitivity analysis was performed to determine whether particular studies could have influenced the heterogeneity or effect size. Therefore, studies were individually excluded to confirm any significant change in effect size for each outcome measure. Any significant difference of one or two studies was reported in the results. An Egger's test and funnel plot are also included to assess potential publication bias.

## 3 Results

### 3.1 Results of the Search

A total of 4636 studies were identified based on the search strategy, with 424 references removed due to database duplication. A total of 4212 studies were screened for abstract/title screening by two independent reviewers, of which 179 were assessed for eligibility. Of the studies screened between the two reviewers, the kappa statistic was 0.87, indicating almost perfect agreement during the selection process. Based on the full‐text screening, 157 studies were excluded for reasons that were not available (33) or because they did not meet the inclusion criteria (124). A total of 22 studies were eligible for data extraction and quality assessment, and all are included in the review. These studies involved healthy, overweight, and obese participants, metabolic syndrome, pregnant, T2DM, and participants with reported dyslipidaemia.

### 3.2 Study Characteristics

Table 2 summarises the population characteristics of each study, including sample size, BMI group of the target population, intervention and control group, dosage and duration of the intervention, adipocytokines measured, study design and key findings. A total of 1299 participants were included in the meta‐analysis. Each study's included population was classified as healthy, overweight, or obese based on the World Health Organization classification of each BMI status. All studies reported the BMI of the intervention and comparison groups. Seven studies included a healthy population, nine included an overweight population, and eleven included an obese population. The primary sources of anthocyanins were pre‐made capsules and extracts, as well as various berries, including blueberries, blackcurrants and bilberries, or a combination of anthocyanin‐rich foods. The dosage of pure anthocyanins ranged from 40 to 700 mg per day. Most studies had an intervention duration of 1 to 24 weeks. Sixteen studies used a placebo as the comparison group, whereas the other six studies involved no intervention or a healthy group as the comparison. Most interventions focused on IL‐10 (*n* = 12) and IL‐8 (*n* = 9), whereas fewer studies investigated IL‐18 (*n* = 6), IFN‐γ (*n* = 6) and resistin (*n* = 6). Most studies observed multiple biomarkers, but this meta‐analysis only considered biomarkers relevant to the topic.

**TABLE 2:** Risk of Bias Assessment based on the Cochrane Collaboration's risk of bias assessment tool from Covidence.

Author/Year	Random sequence allocation	Allocation concealment	Blinding of participants and personnel	Blinding of outcome assessment	Incomplete outcome data	Selective reporting	Other bias	Overall RoB
Aboonabi and Aboonabi (2020)	High	High	High	High	Low	Low	Unclear	High
Arbizu et al. (2023)	Low	Low	High	Unclear	Low	Low	Unclear	Low
Basu et al. (2021)	Low	Low	Low	High	Low	Low	Low	Low
Borda et al. (2024)	Low	Low	Low	Low	Low	Low	Low	Low
Chong et al. (2024)	Low	Low	Low	Low	Low	Low	Low	Low
Cremonini et al. (2022)	Unclear	Low	Low	Unclear	Low	Low	Low	Low
Espinosa‐Moncada et al. (2018)	Low	Low	Low	Unclear	Low	Low	Low	Low
Guo et al. (2020)	Low	Low	Low	Low	Low	Low	Low	Low
Hokayem et al. (2013)	Low	Low	Unclear	Unclear	Low	Low	Low	Low
Karlsen et al. (2007)	Unclear	Unclear	High	High	Low	Unclear	Unclear	High
Kim et al. (2018)	Low	Low	Low	Low	Low	Low	Low	Low
Kuntz et al. (2014)	Low	Low	Low	Low	Low	Low	Low	Low
Lee et al. (2016)	Low	Low	Low	Unclear	Low	Low	Low	Low
Mendes et al. (2021)	Low	High	High	High	Low	Low	Low	Low
Nikbakht et al. (2021)	High	High	High	High	Low	Low	Unclear	High
Ono‐Moore et al. (2016)	Low	Low	Low	Low	Low	Low	Low	Low
Puupponen‐Pimiä et al. (2013)	Low	Unclear	Unclear	Unclear	Low	Low	Unclear	Low
Stote et al. (2017)	Low	Low	Low	Unclear	Low	Low	Low	Low
Tomé‐Carneiro et al. (2012)	Low	Low	Low	Low	Low	Low	Low	Low
Zhang et al. (2020)	Low	Low	Low	Unclear	Low	Low	Low	Low
Zhu et al. (2022)	Low	Low	Low	Low	Low	Low	Low	Low
Zunino et al. (2014)	Unclear	Low	Low	Low	Low	Low	Low	Low

### 3.3 Risk of Bias in Included Studies

A risk of bias assessment was performed on every included study involved in data analysis. Most studies adhered to some form of randomisation or blinding of participants or outcome assessment. In case–control studies or open‐label studies, the risk of selection and detection bias was high, as the intervention or control group may not represent the population from which they were drawn. Single‐blind RCTs had a higher risk of performance bias due to the absence of blinding of participants or personnel and ineffective blinding of outcome data. Several interventions had participants who dropped out due to follow‐up or other reasons during the study. However, they stated that their data was excluded from the results, along with the reasoning for its exclusion. Results indicated as an ‘unclear risk’ were due to inadequate information that prevented evaluation of the totality of the data. An Egger's test for each gene of interest revealed no indication of any publication bias for IL‐8 (*p* = 0.476), IL‐10 (*p* = 0.298), IL‐18 (*p* = 0.230), IFN‐γ (*p* = 0.078) and resistin (*p* = 0.147). Funnel plots were all symmetrical, suggesting no significant overall bias; however, a few studies, including Nikbakht et al. 2021 (IL‐8), Arbizu et al. 2023 (IL‐10), Kim et al. 2018 (IFN‐y), Hokayem et al. 2013 (Resistin), and Basu et al. 2021 (Resistin), were outliers (Appendices 1, 2, 3, 4, 5). Potential causes may arise due to the robustness of the study design, or may be caused by selective reporting by researchers, or may favour studies with statistically significant findings over inconclusive results.

### 3.4 Effects of Anthocyanins on Inflammatory Biomarker Concentrations

**FIGURE 3:**
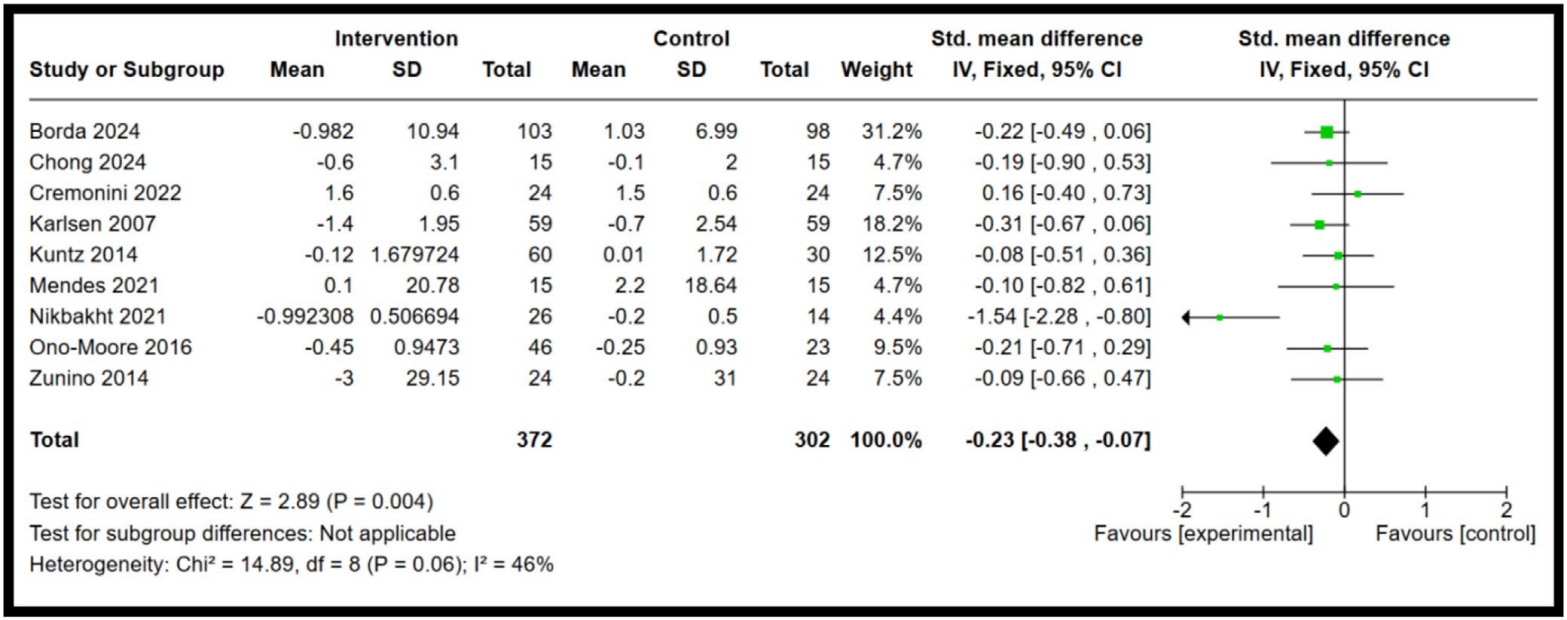
Forest plot for interleukin‐8. Black diamonds represent the pooled effect values. Green squares represent the weight of each study. Horizontal lines represent the 95% confidence intervals. A *p*‐value represented the test for the overall effect; *p* < 0.05 indicated statistical significance. I‐squared measured heterogeneity.

**FIGURE 4:**
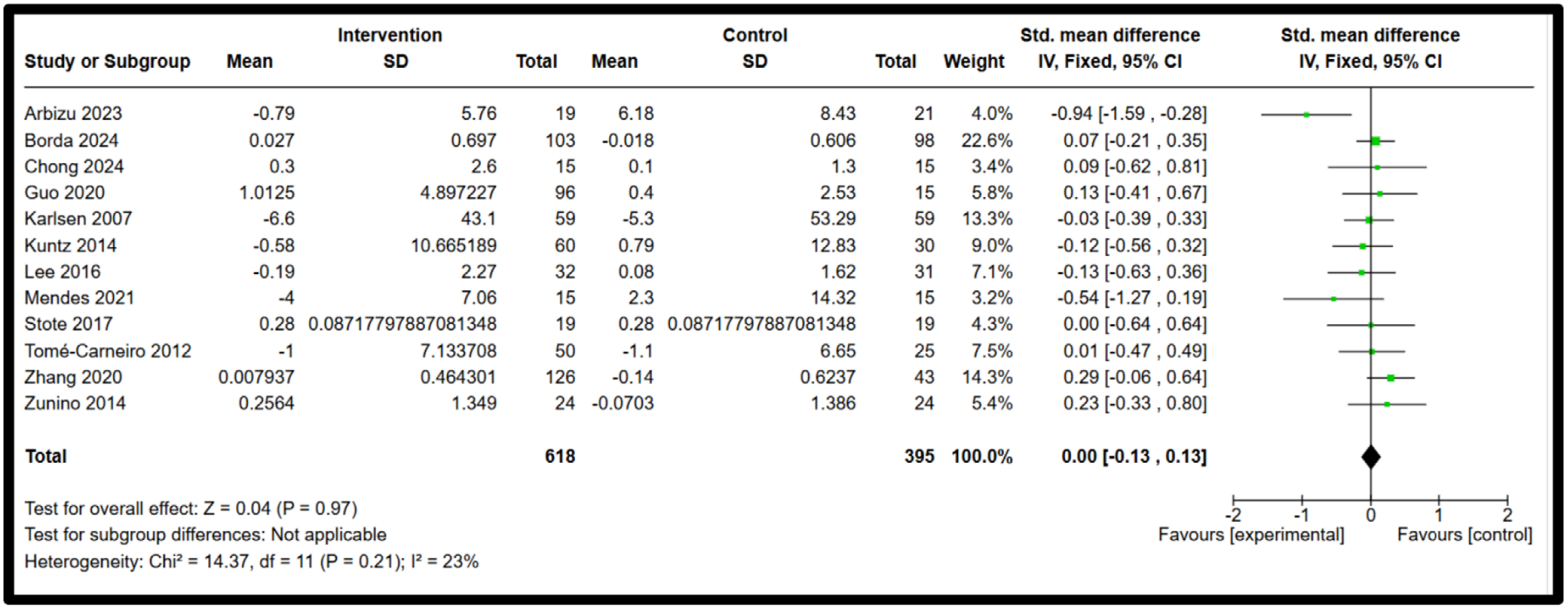
Forest plot for Interleukin‐10. Black diamonds represent the pooled effect values. Green squares represent the weight of each study. Horizontal lines represent the 95% confidence intervals. A *p*‐value represented the test for the overall effect; *p* < 0.05 indicated statistical significance. I‐squared measured heterogeneity.

**FIGURE 5:**
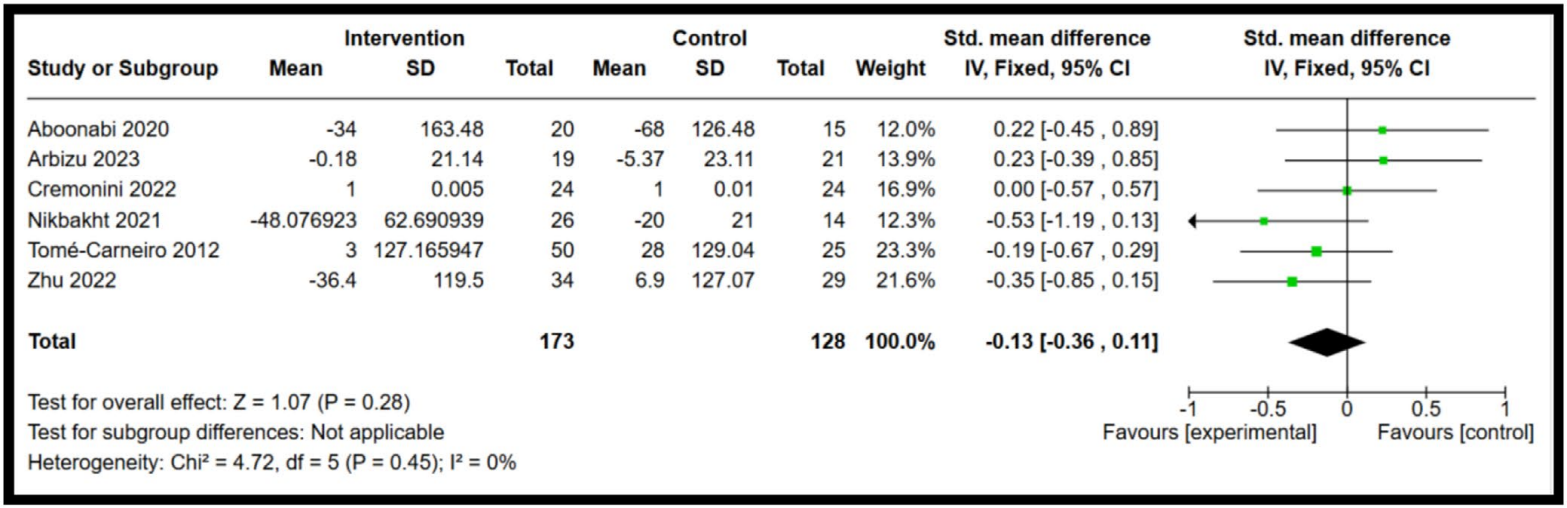
Forest plot for Interleukin‐18. Black diamonds represent the pooled effect values. Green squares represent the weight of each study. Horizontal lines represent the 95% confidence intervals. A *p*‐value represented the test for the overall effect; *p* < 0.05 indicated statistical significance. I‐squared measured heterogeneity.

**FIGURE 6:**
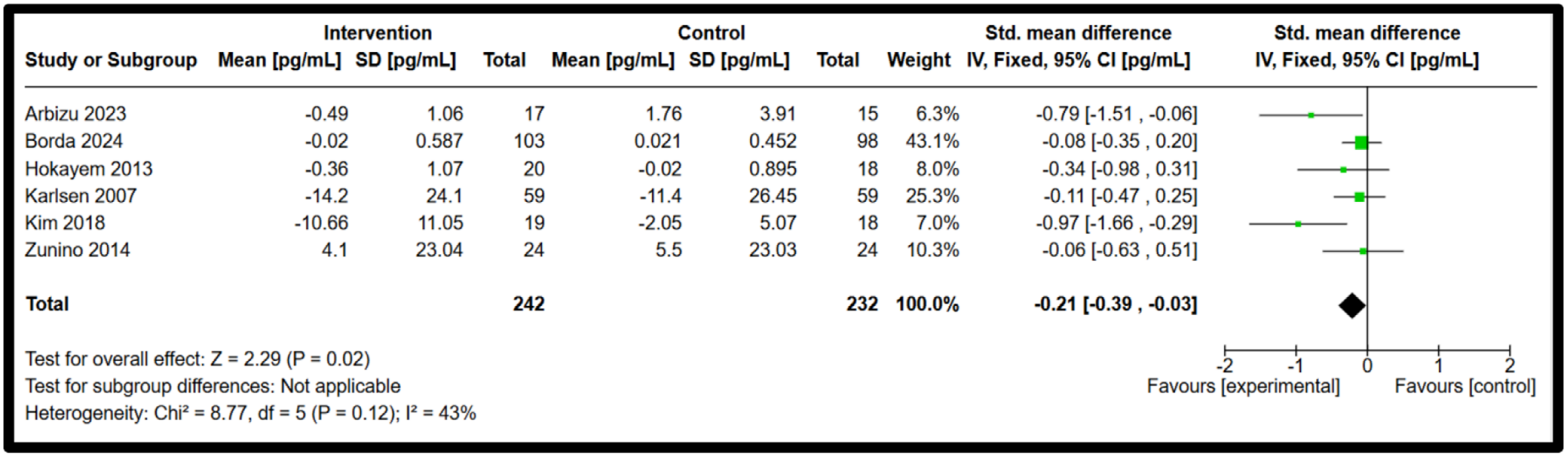
Forest plot for Interferon‐gamma. Black diamonds represent the pooled effect values. Green squares represent the weight of each study. Horizontal lines represent the 95% confidence intervals. A *p*‐value represented the test for the overall effect; *p* < 0.05 indicated statistical significance. I‐squared measured heterogeneity.

**FIGURE 7:**
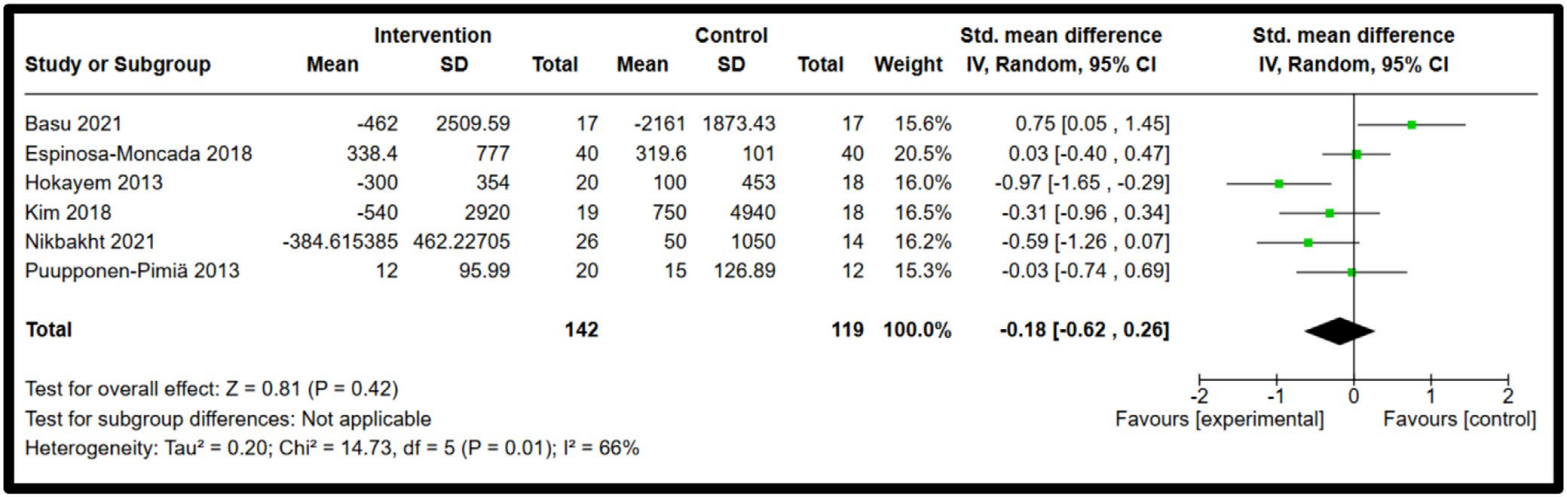
Forest plot for Resistin. Black diamonds represent the pooled effect values. Green squares represent the weight of each study. Horizontal lines represent the 95% confidence intervals. A *p*‐value represented the test for the overall effect; *p* < 0.05 indicated statistical significance. I‐squared measured heterogeneity.

Among the five biomarkers observed, IL‐8 and IFN‐γ showed statistical significance, whereas IL‐10, IL‐18 and resistin showed a decrease in biomarker concentrations, but no significant differences were observed. Forest plots illustrating the pooled effects of anthocyanin supplementation for each biomarker are presented in Figures 3, 4, 5, 6, 7. Nine studies investigated the effect of anthocyanin consumption on plasma IL‐8 levels, in which the overall effect was significant (*p* = 0.004, standardised mean difference (SMD) −0.23, 95%, confidence interval (CI) [−0.38, −0.07]). Similarly, a significant overall effect of IFN‐γ (six studies) was observed in the comparison between the intervention and control groups (*p* = 0.02, SMD = −0.21, 95% CI = [−0.39, −0.03]). This indicates that anthocyanin treatment is associated with decreased levels of IL‐8 and IFN‐γ in a healthy, overweight, and obese population. The I‐squared value was 46% (*p* = 0.06) and 43% (*p* = 0.12), respectively, suggesting moderate heterogeneity and potential inconsistency in the results between the included studies. On the other hand, a meta‐analysis of 12 studies examining IL‐10 levels (*p* = 0.97, SMD 0.00 95% CI [−0.13, 0.13]), 6 studies examining IL‐18 levels (*p* = 0.28, SMD −0.13 95% CI [−0.36, 0.11]) and 6 studies examining resistin levels (*p* = 0.42, SMD −0.18 95% CI [−0.62, 0.26]), found no significant effect. The I‐squared value for resistin was 66% (*p* = 0.01), indicating substantial heterogeneity among the included studies. A random effects model was used for the analysis of resistin concentrations (I‐squared > 50%), whereas the other forest plots used a fixed effect model. Due to the substantial heterogeneity of the resistin studies, a random‐effects model is more appropriate, as heterogeneity between studies is likely influencing the effect size (McKenzie and Veroniki 2024). Effect sizes and heterogeneity remained stable for IL‐8, IL‐10, resistin, and IL‐18 after performing a sensitivity analysis. However, a sensitivity analysis for IFN‐γ heavily influenced the overall effect size, in which two studies were removed that had a total weight of 13.3% (Appendix 6). The effect size is then not significant (*p* = 0.27, SMD = −0.11, 95% CI = −0.30, 0.09), and there is no heterogeneity between the included studies (I‐squared = 0%, *p* = 0.91). This suggests that the results are not robust, and further research is needed to establish significant findings with an increased sample size. Subgroup analyses based on BMI categories (healthy, overweight, and obese) could not be conducted due to the limited studies reporting stratified results. The lack of available studies would have likely resulted in a non‐significant overall effect size, with a reduced number of participants leading to lower statistical power to detect a true effect. Table 3 presents a summary of each measured biomarker, including overall effect size, heterogeneity, and the *p*‐value (Egger's test).

**TABLE 3:** Meta‐analysis for the effects of anthocyanins on inflammatory biomarkers.

Outcome measure	Comparisons (*n*)	Overall effect size	Heterogeneity (I‐squared% %)	Egger's test (*p*)
IL‐8	674	−0.23	46%	0.476
IL‐10	1013	0.00	23%	0.298
IL‐18	301	−0.13	0%	0.230
IFN‐γ	474	−0.21	43%	0.078
Resistin	261	−0.18	66%	0.147

## 4 Discussion

To the best of our knowledge, this is the first meta‐analysis to investigate the impact of anthocyanins on specific inflammatory adipokines (IL‐8, IL‐10, IL‐18, IFN‐γ, resistin) in a healthy, overweight, and obese population. In total, 22 studies were included across 5 biomarkers, in which IL‐8 and IFN‐γ showed statistically significant reductions in plasma concentrations. A sensitivity analysis revealed that excluding two studies resulted in a decrease in effect size, thereby altering the statistical significance of IFN‐γ levels. On the other hand, IL‐10, IL‐18 and resistin showed no considerable importance in the pre‐ and post‐supplementation of anthocyanins. Despite the lack of significance, the findings suggest that anthocyanin supplementation may reduce major inflammatory cytokines that play a substantial role in obesity‐induced inflammation, which supports the hypothesis of this meta‐analysis. Previous meta‐analyses have reported findings of anthocyanins in other inflammatory biomarkers. Sangsefidi et al. performed a meta‐analysis of seven studies that examined the effect of anthocyanins on C‐reactive protein levels, indicating no significant impact (Sangsefidi et al. 2018). On the other hand, Fallah et al. assessed several markers, including CRP, IL‐6, and TNF‐α, of which 32 randomised controlled trials indicated significantly decreased levels for all three (Fallah, Sarmast, Fatehi, and Jafari 2020). The significant findings of multiple inflammatory cytokines, which are highly relevant to the pathogenesis of obesity‐induced inflammation, highlight the potential of anthocyanins to modulate key pro‐inflammatory mechanisms. Comparing these results with the current meta‐analysis provides a more comprehensive understanding of anthocyanins' anti‐inflammatory potential across both more and less frequently studied markers.

Current evidence implies that obesity is directly linked to the primary cause of various chronic diseases, including cardiovascular diseases, diabetes, and certain types of cancer. Prolonged release of pro‐inflammatory adipokines is characteristic of obesity‐linked inflammation, which in turn exacerbates metabolic dysregulation and increases the risk of developing chronic diseases. These mediators are activated by inflamed adipocytes, which trigger an immune response. Circulating levels of pro‐inflammatory cytokines in the serum are significantly higher in obese subjects compared to non‐obese subjects. Several signalling pathways influence the pathogenesis of obesity through the MAPK, PI3K and JAK/STAT signalling pathways (Wen et al. 2022). IL‐8 is a major mediator in the inflammatory response, inducing neutrophil chemotaxis and stimulating neutrophil transmigration (Takami et al. 2002). IL‐8 promotes cancer cell invasion and atherosclerosis by activating the PI3K/Akt pathway, making it a potential therapeutic target for obesity (Takami et al. 2002). The statistical significance of the reduction in IL‐8 levels from anthocyanin supplementation suggests that anthocyanins can directly affect the signalling mechanisms involved in inflammation and cancer proliferation. Anthocyanins induce apoptosis in cancer cells by suppressing the Akt/mTOR signalling pathway and the PI3K/Akt signalling pathway, which are linked to the promotion of IL‐8 production (Hennessy et al. 2005; Wen et al. 2022).

Similarly, IL‐18 also plays a role in the pathogenesis of metabolic diseases and is suspected to induce insulin resistance, as evidenced by studies investigating IL‐18 levels in diabetic obese patients (Ahmad et al. 2017; Bruun et al. 2007). However, the understanding of the link between anthocyanins and IL‐18, as well as their relationship to obesity, is minimal. Based on a meta‐analysis, there is no statistical significance in reducing IL‐18 levels. A study even suggested that adipose tissue expression of IL‐18 may be increased in obesity, but not affected by weight loss, indicating that the mechanism of action is related to insulin resistance (Bruun et al. 2007). Further research would be needed on the mechanism of action of IL‐18 in an obese population, as well as its long‐term effects, given that the average intervention time was only 4 weeks. Studies have also examined the effect of anthocyanin supplementation on the concentration of resistin, another inflammatory mediator that promotes insulin resistance by increasing glucose production in the liver and inhibiting the differentiation of preadipocytes into adipocytes (Wolf 2004). Resistin is also linked to driving chronic inflammation and diabetes. Activation of the NF‐kB pathway is linked to increased oxidative stress and amplifies the inflammatory status. Six studies have investigated the impact of anthocyanin supplementation on serum resistin concentrations in humans; however, no significance could be identified between the studies. The link between anthocyanin supplementation and resistin levels is not well established in humans; therefore, more human clinical trials are required. One meta‐analysis investigated the effect of dietary anthocyanins on glucose metabolism and found a statistical significance on resistin levels; however, due to the small number of studies included, there is limited statistical power, and the effect size can be considered insignificant (Fallah, Sarmast, and Jafari 2020).

This meta‐analysis revealed a significant effect of dietary and supplement anthocyanins on decreasing levels of IFN‐γ compared to a control group. NK cells, CD4+ Th1 cells, and CD8+ T cells secrete IFN‐γ, which enhances antigen recognition by antigen‐presenting cells like macrophages and dendritic cells (Huang et al. 2022; Sica and Mantovani 2012). This leads to the polarisation of macrophages into the M1 phenotype and elevates the production of pro‐inflammatory cytokines (Huang et al. 2022; Sica and Mantovani 2012). IFN‐γ is overexpressed in adipose tissue of obese subjects, due to the excess nutrient conditions that result in a pro‐inflammatory state and oxidative stress. Anthocyanins have been observed to modulate immune responses by interrupting the activity of specific immune cells and inhibiting the phosphorylation of STAT1, which is a significant transcription factor in the IFN‐γ signalling pathway (Kowalczyk et al. 2024; Roth et al. 2014). No meta‐analysis has focused on the effects of anthocyanins on IFN‐γ levels; however, this meta‐analysis suggests that anthocyanins have a significant modulatory effect on IFN‐γ, consistent with previous studies.

One of the primary objectives of this meta‐analysis was to investigate the potentially significant effect of anthocyanins on anti‐inflammatory cytokines, such as IL‐10. It primarily inhibits the production of pro‐inflammatory adipokines and enhances regulatory T‐cell function, preventing damage to adipocytes and maintaining balanced tissue homeostasis (Charles et al. 2011; Iyer and Cheng 2012). Several studies have investigated the effects of anthocyanin supplementation to determine if there is a significant reduction, which could indicate their potential role in enhancing anti‐inflammatory modulators. The summary of the included studies concludes that there is currently no significant correlation between levels of IL‐10 and the consumption of anthocyanins. Thus, further studies will be necessary to investigate the gene expression of IL‐10 in overweight and obese individuals to better understand whether improved levels lead to long‐term fat loss.

This meta‐analysis has several strengths. The targeting of relatively underexplored inflammatory markers that have not been analysed under a meta‐analysis format addresses a clear gap in the current literature. It contributes to a more nuanced understanding of the anti‐inflammatory effects of anthocyanins and promotes future research that applies these biomarkers to improve anthocyanin‐based interventions. The inclusion of all BMI populations enables the findings to be applied to a diverse range of participants, thereby increasing the generalizability of the results. The literature search was comprehensive, screening a large number of studies to ensure that all relevant evidence was identified and minimising the chance of missing significant data. The study quality and risk of bias were critically evaluated in each study, thereby improving the reliability of the data. The inter‐rater reliability was also assessed, with a high Cohen's kappa statistic, ensuring that the study selection and screening process were consistent. This strengthened the validity and generalizability of the research findings and meta‐analysis results.

There are a few limitations that the presenting meta‐analysis includes. First, the two studies used were not RCTs or trials that excluded some form of randomisation and blinding that would reduce bias and confounding factors. Several studies indicated ‘high’ or ‘unclear’ levels of blinding or randomisation, which may limit the certainty of the conclusions. Only a handful of studies could be included in examining IL‐18, IFN‐γ and resistin, ultimately due to the lack of studies available that would fit the eligibility criteria. The small number of studies found also reduced the reliability of the funnel plot, as the low power of the tests is too low to distinguish chance from fundamental asymmetry. Based on a sensitivity analysis, the effect size of IFN‐γ was heavily influenced by the weight of the two studies, suggesting that the overall effect may not be robust. Some of the included trials had sample sizes of less than 20 participants, making them more susceptible to bias and limiting the precision of individual study estimates. A sensitivity analysis evaluated the impact of these trials on the overall effect size, revealing no significant change; however, the inclusion of more adequately powered studies would provide greater confidence in the estimated effects. Another major limitation is the inclusion of all types of health status participants in the analysis, rather than focusing on just obesity subjects. Previous research has shown that the effects of anthocyanins may vary depending on the participants' health status (Ockermann et al. 2021). In individuals with obesity or metabolic disorders, anthocyanins have been shown to improve insulin sensitivity and reduce inflammation. In contrast, the benefits may be more subtle in healthy participants, as they already have well‐functioning metabolic and physiological systems. However, due to the limited number of available studies, it was necessary to include all BMI groups to achieve statistical significance in the overall effect size and *P*‐value. This approach also enables a more comprehensive understanding of the potential benefits of anthocyanin supplementation across diverse populations. A subgroup analysis would facilitate a more thorough understanding of the relationship between anthocyanin supplementation and BMI; however, given the limited number of available studies, it is likely to yield no significant findings. Using BMI as the sole indicator for population criteria is a major limitation in terms of accuracy for individuals and specific populations. It is not a direct measure of body fat or adiposity, and does not assess the concomitant presence of comorbid conditions or disease risks(Callahan 2023). Assessing other body measurements, such as waist circumference and waist‐to‐hip ratio, is a more accurate indicator of adiposity and obesity‐related risk (Sweatt et al. 2024). Studies involving dietary foods or extracts often contain other components that may influence the effects of anthocyanins and may not accurately represent the overall effect size in each biomarker; therefore, they are a potential confounding factor. Despite this, all included studies quantified the amount of anthocyanins in the intervention and were at a high enough dose to consider for a meta‐analysis investigation. However, the different forms of anthocyanins provided, including food extracts and whole foods, can influence the bioavailability and efficacy of anthocyanins. Several factors can influence the bioavailability of anthocyanins in the gastrointestinal tract, including their chemical structure, molecular size, and their combination with other compounds and foods, which affect the absorption of anthocyanins and their impact on inflammatory markers (Ayvaz et al. 2022). Different bioactive metabolites can also heavily influence the anti‐inflammatory effectiveness of anthocyanins, with some metabolites demonstrating greater efficacy in reducing markers such as IL‐6, TNF‐α and VCAM‐1 (Gao et al. 2025). The duration of the intervention can also significantly impact the effectiveness of anthocyanins. The included studies had a vast range from 1 week to 24 weeks, making it unclear whether the duration of the trial is sufficient to produce consistent findings in inflammatory cytokines. One meta‐analysis found that the duration of the intervention can significantly impact the effectiveness of anthocyanins, with prolonged periods associated with improvements in inflammatory biomarkers (Kapoor et al. 2023). Future enhancements would include performing a subgroup analysis on the intervention duration to determine whether short or long‐term anthocyanin intervention has differential effects on specific inflammatory cytokines. The addition of a subgroup analysis to separate randomised controlled trials from non‐randomised trials would help determine any potential confounding factors present in the non‐randomised trials.

Overall, this meta‐analysis of 22 high‐quality clinical trials revealed significant reductions in the inflammatory biomarkers IL‐8 and IFN‐γ, indicating that anthocyanin supplementation or consumption of anthocyanin‐rich foods has beneficial, anti‐inflammatory effects in healthy, overweight and obese populations. However, IL‐10, IL‐18 and resistin did not display significant changes, as did IFN‐γ within a sensitivity analysis, suggesting that anthocyanin supplementation may be ineffective for specific inflammatory pathways or requires further research to explore its mechanism of action. Future clinical trials should investigate the dose–response range of anthocyanins to determine an optimal concentration that produces significant anti‐inflammatory effects. Studies should also subgroup multiple populations, including healthy, overweight, and obese subjects, which may help identify biological variability influencing their response to anthocyanin supplementation.

## Author Contributions

**Madiha Ajaz:** methodology, software, conceptualization. **Alessandro Young:** writing – original draft, methodology, validation, visualization, conceptualization, data curation, software, formal analysis, investigation. **Natalie Shilton:** supervision, writing – review and editing, study design, conceptualization. **Lada Vugic:** supervision, writing – review and editing, conceptualization.

## Ethics Statement

This study does not involve any human or animal testing.

## Conflicts of Interest

The authors declare no conflicts of interest.

## Supporting information


**Table S1:** PRISMA checklist.

## Data Availability

The data supporting the findings of this study are available within the article. Raw data that support the findings of this study are available from the corresponding author upon reasonable request.

## Appendix 1

Funnel Plot Illustrating the Publication Bias of Primary IL‐8 Studies.

## Appendix 2

Funnel Plot Illustrating the Publication Bias of Primary IL‐10 Studies.

## Appendix 3

Funnel Plot Illustrating the Publication Bias of Primary IL‐18 Studies.

## Appendix 4

Funnel Plot Illustrating the Publication Bias of Primary IFN‐y Studies.

## Appendix 5

Funnel Plot Illustrating the Publication Bias of Primary Resistin Studies.

## Appendix 6

Sensitivity Analysis of the IFN‐γ Forest Plot.
